# Effects of accumulated exercise on the stiffness and hemodynamics of the common carotid artery

**DOI:** 10.3389/fphys.2024.1348811

**Published:** 2024-02-26

**Authors:** Haibin Liu, Bingyi Shen, Zusheng Li, Chundong Xue, Hongling Zhao, Xin Pan, Dong Xu

**Affiliations:** ^1^ School of Sport and Health Sciences, Dalian University of Technology, Dalian, Liaoning, China; ^2^ Department of Neurology, Central Hospital of Dalian University of Technology, Dalian, Liaoning, China; ^3^ School of Bioengineering, Dalian University of Technology, Dalian, Liaoning, China; ^4^ School of Biomedical Engineering, Faculty of Medicine, Dalian University of Technology, Dalian, Liaoning, China

**Keywords:** accumulated exercise, continuous exercise, common carotid artery, arterial stiffness, hemodynamics

## Abstract

**Purpose:** This research aims to study and compare the effects of moderate-intensity continuous exercise and accumulated exercise with different number of bouts on common carotid arterial stiffness and hemodynamic variables.

**Methods:** Thirty healthy male adults were recruited to complete four trials in a randomized crossover design: no-exercise (CON); continuous exercise (CE, 30-min cycling); accumulated exercise including two or three bouts with 10-min rest intervals (AE15, 2 × 15-min cycling; AE10, 3 × 10-min cycling). The intensity in all the exercise trials was set at 45%–55% heart rate reserve. Blood pressure, right common carotid artery center-line velocity, and arterial inner diameter waveforms were measured at baseline and immediately after exercise (0 min), 10 min, and 20 min.

**Results:** 1) The arterial stiffness index and pressure–strain elastic modulus of the CE and AE15 groups increased significantly at 0 min, arterial diameters decreased in AE15 and AE10, and all indicators recovered at 10 min. 2) The mean blood flow rate and carotid artery center-line velocity increased in all trials at 0 min, and only the mean blood flow rate of AE10 did not recover at 10 min. 3) At 0 min, the blood pressure in all trials was found to be increased, and the wall shear stress and oscillatory shear index of AE10 were different from those in CE and AE15. At 20 min, the blood pressure of AE10 significantly decreased, and the dynamic resistance, pulsatility index, and peripheral resistance of CE partially recovered.

**Conclusion:** There is no significant difference in the acute effects of continuous exercise and accumulated exercise on the arterial stiffness and diameter of the carotid artery. Compared with continuous exercise, accumulated exercise with an increased number of bouts is more effective in increasing cerebral blood supply and blood pressure regulation, and its oscillatory shear index recovers faster. However, the improvement of blood flow resistance in continuous exercise was better than that in accumulated exercise.

## 1 Introduction

In the past few decades, the incidence and prevalence of congenital and acquired cardiovascular diseases (CVDs) have increased around the world. With the aging of the population, the burden of CVDs will continue to increase (Andersson and Vasan, 2018). Early control of CVD risk factors can effectively prevent the occurrence and development of cardiovascular events. The common carotid artery is the main artery trunk of the head, and carotid arterial stiffness is a subclinical marker associated with cardio-cerebrovascular diseases and mortality (Saz-Lara et al., 2021). The changes in the structure and function of common carotid arteries are highly correlated with atherosclerosis, coronary ischemia, and stroke (Yacoub et al., 2014). Hemodynamic variables, including blood pressure (BP), pulsatility index (PI), blood flow velocity, blood flow-induced wall shear stress (WSS), and oscillatory shear index (OSI), induce structural and functional changes in the arteries (Tinken et al., 2010). Hemodynamics changes are key to reflect and regulate cardiovascular health.

Exercise-induced direct hemodynamics impacts on the artery wall significantly reduce cardiovascular risk and improve arterial stiffness (Green et al., 2017). The US Physical Activity Guidelines have recommended that physical activity can be accumulated in shorter bouts across the day, totaling the recommended amount of physical activity for health (Garber et al., 2011). Considering that the risk of cardiovascular complications increases during vigorous physical exertion, especially for persons who have latent or documented coronary artery disease and are habitually sedentary, multi-bout accumulated exercise is safer and more acceptable and hence can be used as an effective strategy to enhance exercise compliance and reduce sports fatigue. Many studies have reported that compared with continuous exercise, accumulated exercise has a more significant effect on many aspects of the human body, such as reducing postprandial blood glucose response (Gouldrup and Ma, 2021), regulating blood pressure (Bhammar et al., 2012), and reducing arterial stiffness (Zheng et al., 2015).

The duration and number of exercise bouts affect the acute effects of accumulated exercise on arterial stiffness. Previous studies related to accumulated exercise mostly focused on the effect of different interval times of accumulated exercise. It has been summarized that, at least as to cardiovascular risk factors, only when the residual effect from the previous exercise bout exists, the acute exercise effect may accrue in the next exercise bout (Thompson et al., 2001). Accumulated exercise with a shorter, as opposed to a longer, rest interval between exercise bouts elicited more pronounced neuroendocrine (Ronsen et al., 2002) and augmented metabolic stress (Ronsen et al., 2004). However, only a few studies have addressed the effects of different numbers of bouts of accumulated exercise on human health, especially on arterial stiffness and hemodynamics.

The purpose of this study is to investigate the effects of moderate-intensity accumulated exercise with different numbers of bouts and the same interval time on arterial stiffness and more comprehensive hemodynamic variables in healthy young men. We hypothesize that the acute benefits of accumulated exercise with a shorter exercise bout duration can be compensated by increasing the number of exercise bouts, thereby maintaining the superior effect of accumulation on arterial stiffness and hemodynamics.

## 2 Methods

### 2.1 Subjects

Thirty healthy male undergraduates aged 18.76 ± 1.81 were recruited in this study (Table 1). The inclusion criteria specified men who were not involved in any regular physical activity (exercise less than three times a week and 30 min each time for the previous 6 months). They were not obese and did not smoke and did not have a history of heart disease, hypertension, or other diagnosed cardiovascular or metabolic diseases. Subjects were instructed to avoid vigorous physical activity and consumption of alcohol, medication, and caffeine 24 h before the experiment and to fast overnight (>12 h). On the day of the experiment, a baseline test was performed after a period of quiet rest for at least 10 min.

**TABLE 1:** Baseline characteristics of the subjects (n = 30).

	Value (mean ± SD)
Age (years)	18.76 ± 1.81
Height (cm)	173.42 ± 6.14
Weight (kg)	61.69 ± 6.30
Body mass index (kg/m^2^)	20.56 ± 2.30
Rest heart rate (beats/min)	69.04 ± 6.78

### 2.2 Experimental design

The pre-experiment was performed to determine the interval time of the accumulated exercise trials. The results indicated that after a single bout of 10-min moderate-intensity cycling, almost all the hemodynamic variables in this study were returned to the baseline level 10 min after the completion of the exercise. Therefore, in order to ensure that the acute hemodynamics response induced by the previous bout of exercise did not completely recover before the next bout of exercise, the interval time of the accumulated exercise trial was set as 10 min.

Each subject participated in four trials in random order, which were the non-exercise trial (CON), 30-min continuous exercise trial (CE), accumulated exercise in two 15-min bouts with 10-min intervals (AE15), and accumulated exercise in three 10-min bouts with 10-min interval trial (AE10). In order to avoid interference between the trials, the trials were conducted with an interval of 7 days.

**FIGURE 1:**
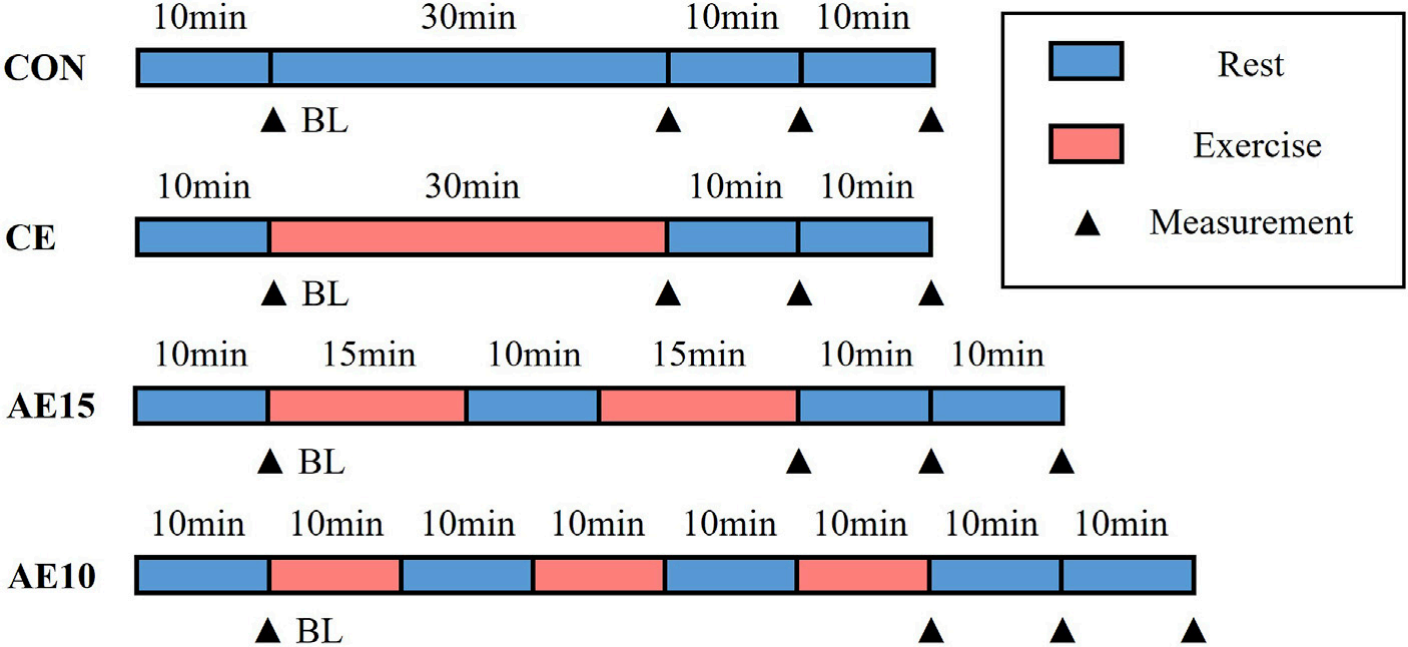
Exercise intervention protocol. Four experimental trials include a non-exercise trial (CON), a 30-min continuous exercise trial (CE), accumulated exercise in two 15-min bouts with 10-min intervals (AE15), and accumulated exercise in three 10-min bouts with 10-min intervals (AE10). Time points of measurements are baseline (BL), immediately after exercise (0 min), 10 min, and 20 min.

The experiment protocol is shown in Figure 1. In the CON trial, after the first baseline (BL) assessment, the subjects were placed in a supine position quietly for 30 min, and the subsequent three assessments were performed at 0, 10, and 20 min after the end of 30-min supine position assessment. In the CE trial, following the BL assessment, the subjects exercised on a power bicycle for 30 min continuously and were immediately restored to the supine position, and the assessments were repeated the same as in the CON trial. In the AE15 trial, the 30-min exercise was divided into two 15-min bouts with one 10-min interval. For the AE10 trial, the 30-min exercise was divided into three 10-min bouts with two 10-min intervals, and the assessment of AE15 and AE10 was the same as in the CON trial.

All acute cycling exercise interventions were conducted on a power bicycle (Powermax-VIII, Combi Wellness, Japan) with a fixed workload set to 3 kp. The intensity of exercise was determined by the heart rate reserve (HRR) method. The targeted heart rates were calculated by using the Karvonen formula, namely, target heart rate = (220 − age − resting heart rate) × exercise intensity (%) + resting heart rate. The heart rate of the participant was monitored by using an ear-clip heart rate sensor embedded in the power bike. During the exercise, the heart rate is displayed on the screen of the power bicycle in real-time. The cycling speed was monitored and adjusted by the participants to keep their heart rate within the target heart rate range.

### 2.3 Measurements and calculation of local hemodynamics

**FIGURE 2:**
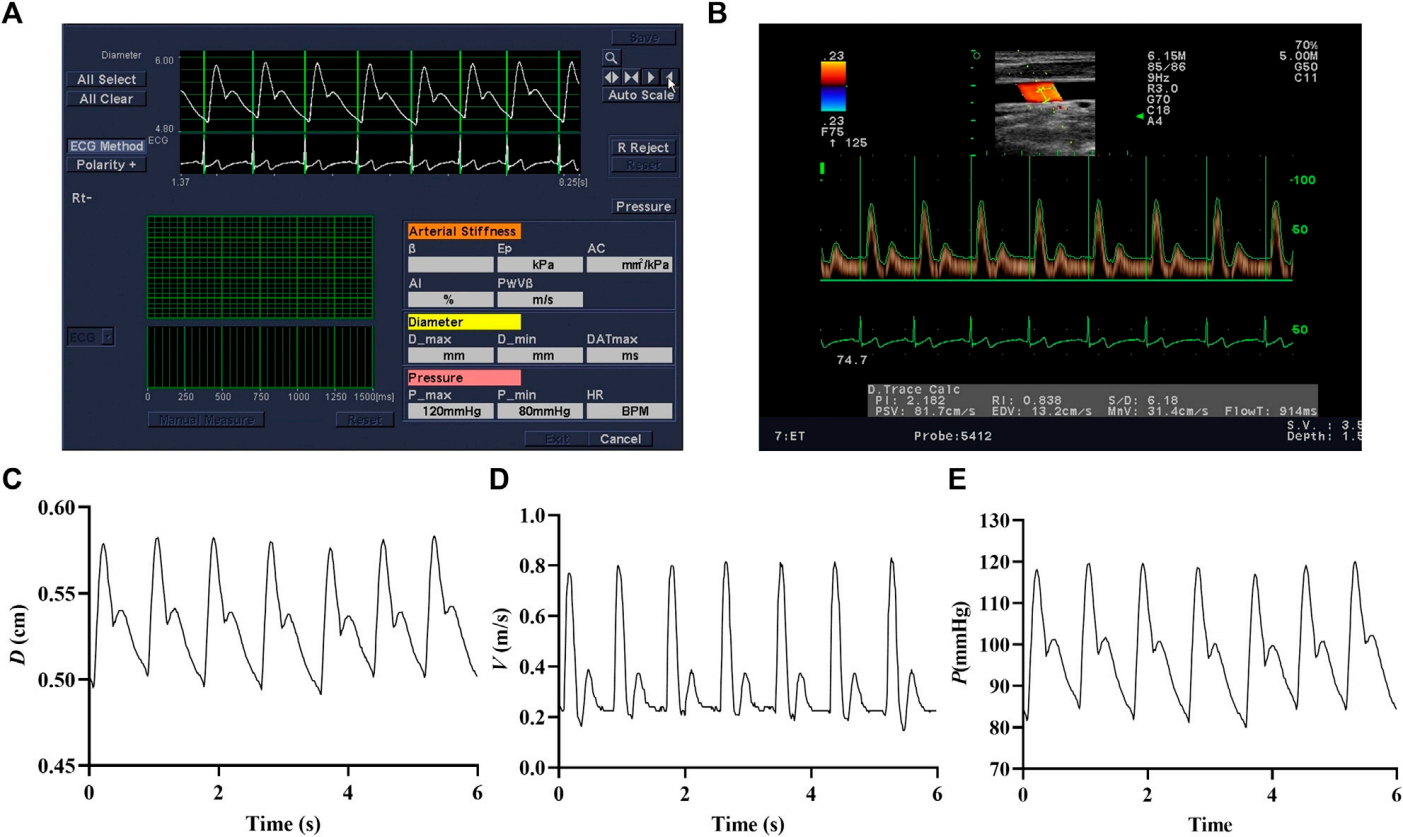
Images of arterial diameter **(A)** and center-line velocity waveforms **(B)**. The extracted arterial diameter **(C),** center-line velocity waveforms **(D),** and calibrated carotid blood pressure waveforms **(E).**

The arterial inner diameter and center-line blood flow velocity waveforms of the right common carotid artery were measured by color Doppler ultrasound (Prosound Alpha 7, Aloka, Japan). In the meantime, the heart rate, brachial systolic pressure, and diastolic pressure were recorded on the left arm in triplicate using a cuff-type electronic manometer (HEM-7136, Omron, Japan). According to the method described in detail in our previous study (Liu et al., 2018), we measured the arterial inner diameter and center-line blood flow velocity waveforms of the right common carotid artery and brachial pressure. The detected arterial diameter waveforms (Figure 2A) and center-line velocity waveforms (Figure 2B) were saved only as images. The self-compiled program in MATLAB was used to extract blood vessel diameter (Figure 2C) and center-line velocity waveforms (Figure 2D). Heart rate signals were used to synchronize the diameter and center-line velocity waveforms (beat-to-beat recording), and then the carotid artery blood pressure waveform (Figure 2E) was calibrated using brachial pressure and arterial diameter waveforms.

After getting the waveforms of the arterial inner diameter, center-line blood flow, and calibrated carotid artery blood pressure, we followed the methods and calculation formulas of Liu et al. (2018); Shen et al. (2020) to calibrate and calculate local apparent arterial stiffness and hemodynamic variables (detailed methods and formulas are in the Supplementary Material). Detailed hemodynamic variables include the blood pressure (BP), flow rate (*Q*), apparent elastic modulus (*E*_p_), apparent stiffness index (*β*), wall shear stress (WSS), oscillatory shear index (OSI), pulsatility index (PI), dynamic resistance (DR), and peripheral resistance (PR).

### 2.4 Data processing and statistical analysis

All hemodynamic variables’ calculation formulas were programmed by MATLAB (The MathWorks R2020a, Inc.). The waveforms of wall shear stress and velocity in the equation were expanded by Fourier series. All data were presented as the mean ± SD. Two-way analysis of variance (ANOVA) was used to examine the effects of different exercise patterns on arterial stiffness and hemodynamic variables across four assessment time points (BL and 0, 10, and 20 min). The level of statistical significance was set at *p* < 0.05.

## 3 Results

### 3.1 Effects of different exercise patterns on the elastic function of the carotid artery

**FIGURE 3:**
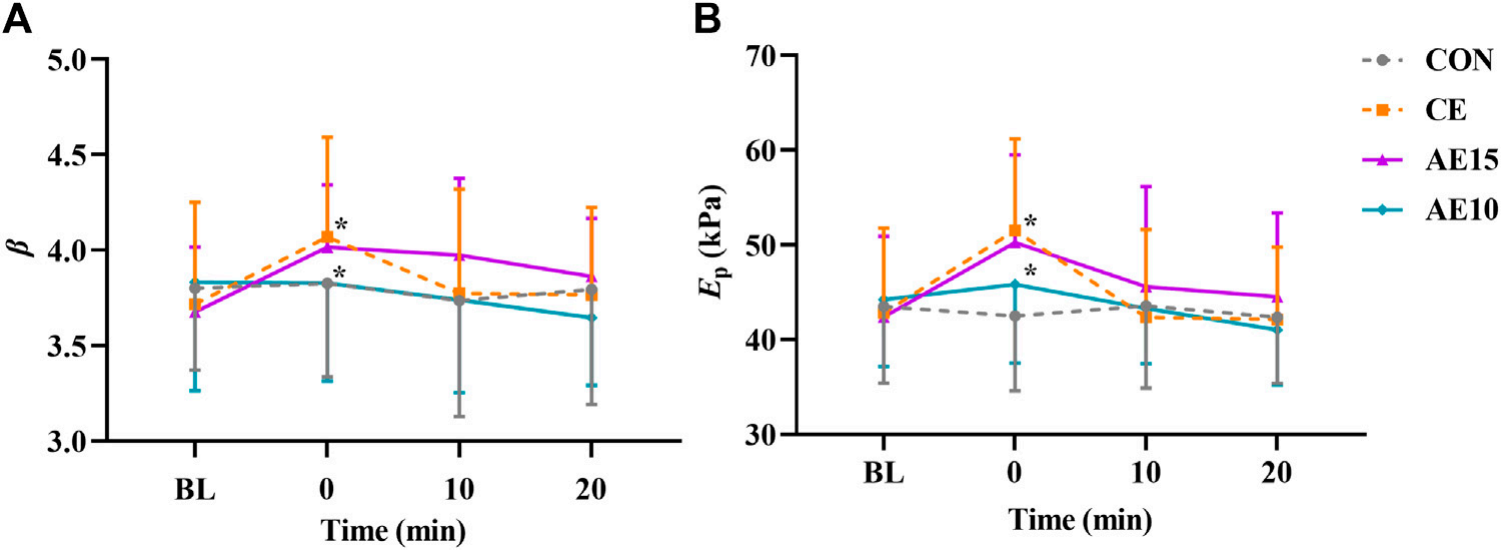
Acute effects of different exercise patterns on elastic function. **(A)** Arterial stiffness index (*β*). **(B)** Pressure–strain elastic modulus (*E*_p_). *p < 0.05 vs BL.

As shown in Figures 3A, B, the arterial stiffness index (*β*) and pressure–strain elastic modulus (*E*_p_) in CE and AE15 were increased significantly immediately after exercise (0 min). There was no significant change in AE10.

**FIGURE 4:**
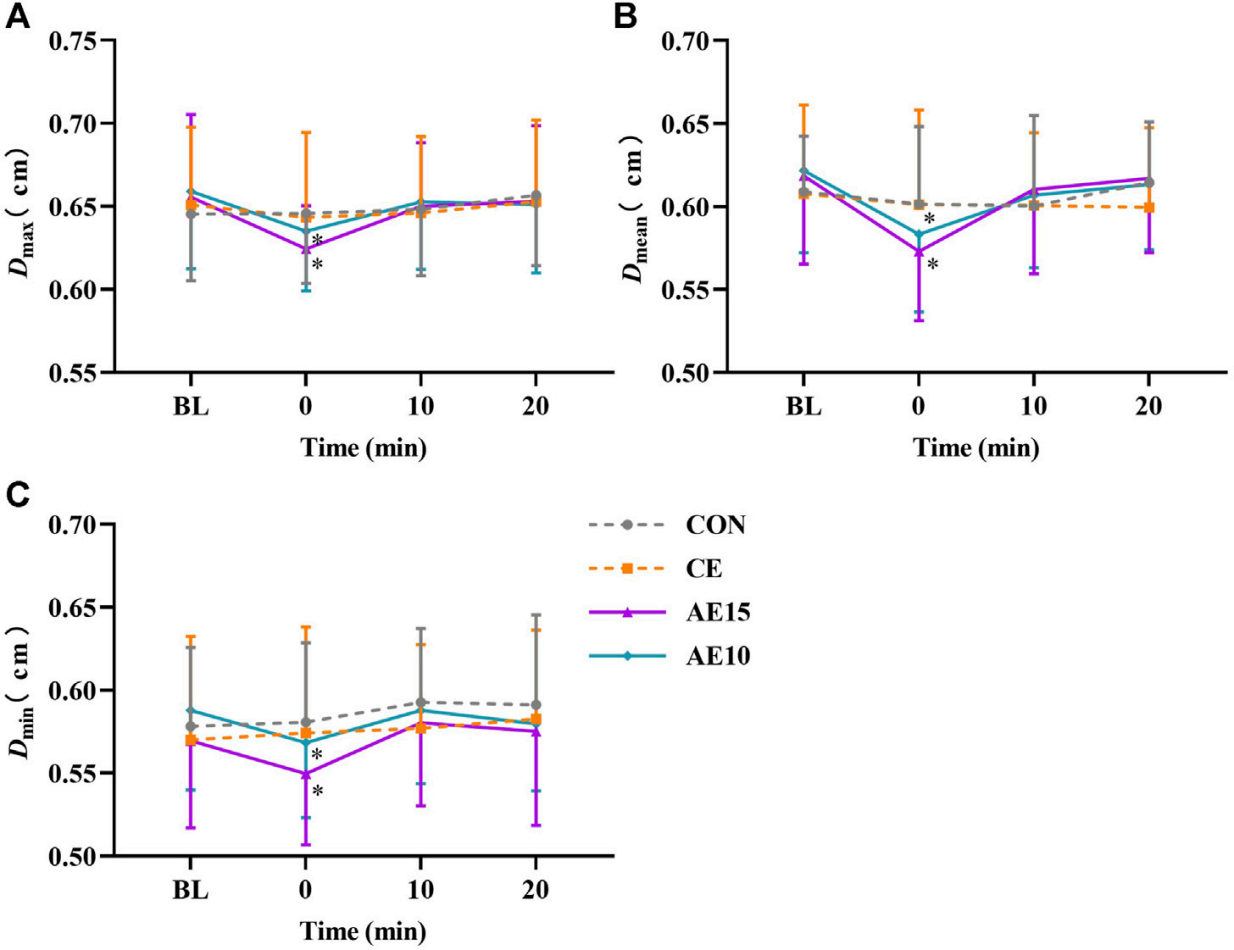
Acute effects of different exercise patterns on carotid artery diameters (D). **(A)** Maximal artery diameters (*D*_max_). **(B)** Mean artery diameters (*D*_mean_). **(C)** Minimal artery diameters (*D*_min_). *p < 0.05 vs BL.

Figures 4A–C indicate the changes in maximal, mean, and minimal carotid diameters (*D*) during different exercise patterns. The representative arterial diameter waveforms of each trial are shown in Supplementary Figure S1. The maximal, mean, and minimal diameters of AE15 and AE10 were decreased significantly at 0 min, and there was no significant change in CE.

### 3.2 Effects of different exercise patterns on the blood supply to the brain

Table 2 and Table 3 shows that the maximum and mean blood flow rate (*Q*_max_ and *Q*_mean_) and center-line velocity (*V*_ma*x*_ and *V*_mean_) of AE10 increased most significantly at 0 min. At 10 min, the *Q*_max_ *, Q*_mean_, and *V*_max_ of AE10 were still significantly higher than those at the BL. The minimum blood flow (*Q*_min_) of all exercise trials switched from forward to reverse, with a significant increase in reverse flow, particularly in the AE10 trial. The representative center-line velocity waveforms of each trial are shown in Supplementary Figure S2.

**TABLE 2:** Effects of different accumulated exercise patterns on common carotid arterial flow rate.

	CON	CE	AE15	AE10
*Q*_max_ (ml/s)				
BL	13.81 ± 0.91	13.76 ± 1.33	13.54 ± 0.73	13.78 ± 1.70
0 min	13.67 ± 1.65	18.00 ± 1.42^*ac^	13.94 ± 1.44^*a^	18.11 ± 2.85^*ac^
10 min	13.66 ± 1.93	15.35 ± 1.56^*a^	14.41 ± 1.18	16.51 ± 3.11^*ac^
20 min	13.58 ± 1.12	14.47 ± 1.51	13.97 ± 0.97	13.43 ± 1.86
*Q*_mean_ (ml/s)				
BL	4.06 ± 0.49	4.07 ± 0.63	4.00 ± 0.56	4.10 ± 0.27
0 min	4.16 ± 0.64	5.26 ± 0.61^*a^	5.05 ± 0.45^*a^	5.37 ± 0.48^*a^
10 min	4.17 ± 0.80	4.20 ± 0.78	4.31 ± 0.74	4.65 ± 0.64^*a^
20 min	4.07 ± 0.54	4.20 ± 0.81	4.13 ± 0.50	4.15 ± 0.60
*Q*_min_ (ml/s)				
BL	0.41 ± 0.71	0.39 ± 0.47	0.42 ± 0.53	0.43 ± 0.62
0 min	0.44 ± 0.51	−2.49 ± 1.21^*a^	−2.37 ± 0.76^*a^	−1.70 ± 1.20^*abc^
10 min	0.49 ± 0.63	−1.24 ± 1.02^*a^	−0.07 ± 1.48^b^	0.08 ± 0.66^b^
20 min	0.44 ± 0.47	0.02 ± 0.67	0.18 ± 0.60	0.44 ± 0.52

Values are the mean ± SD. *Q*_max_: maximal flow rate; *Q*_mean_: mean flow rate; *Q*_min_: minimal flow rate. ‘–’: Blood flow retrograde; *: *p* < 0.05 vs. BL; a: *p* < 0.05 vs. CON; b: *p* < 0.05 vs. CE; c: *p* < 0.05 vs. AE15.

**TABLE 3:** Effects of different accumulated exercise patterns on common carotid center-line velocity.

	CON	CE	AE15	AE10
*V*_max_ (m/s)				
BL	0.71 ± 0.09	0.71 ± 0.11	0.72 ± 0.09	0.72 ± 0.08
0 min	0.70 ± 0.05	0.92 ± 0.17^*a^	0.94 ± 0.17^*a^	1.05 ± 0.16^*abc^
10 min	0.68 ± 0.07	0.73 ± 0.13	0.74 ± 0.11	0.80 ± 0.09^*a^
20 min	0.69 ± 0.08	0.73 ± 0.12	0.71 ± 0.09	0.72 ± 0.11
*V*_mean_ (m/s)				
BL	0.30 ± 0.05	0.30 ± 0.05	0.29 ± 0.04	0.30 ± 0.05
0 min	0.30 ± 0.05	0.38 ± 0.06^*a^	0.38 ± 0.06^*a^	0.39 ± 0.06^*a^
10 min	0.28 ± 0.05	0.30 ± 0.05	0.31 ± 0.04	0.31 ± 0.05
20 min	0.27 ± 0.04	0.30 ± 0.05	0.31 ± 0.05	0.29 ± 0.05
*V*_min_ (m/s)				
BL	0.14 ± 0.07	0.15 ± 0.05	0.13 ± 0.05	0.14 ± 0.06
0 min	0.14 ± 0.06	0.07 ± 0.08^*^	0.09 ± 0.09^*^	0.09 ± 0.08^*^
10 min	0.14 ± 0.06	0.08 ± 0.08^*^	0.10 ± 0.08	0.10 ± 0.07
20 min	0.14 ± 0.05	0.09 ± 0.07^*^	0.11 ± 0.08	0.11 ± 0.07

Values are the mean ± SD. *V*_max_: maximal center-line velocity; *V*_mean_: mean center-line velocity; *V*_min_: minimal center-line velocity. *: *p* < 0.05 vs. BL; a: *p* < 0.05 vs. CON; b: *p* < 0.05 vs. CE; c: *p* < 0.05 vs. AE15.

### 3.3 Effects of different exercise patterns on hemodynamics

**FIGURE 5:**
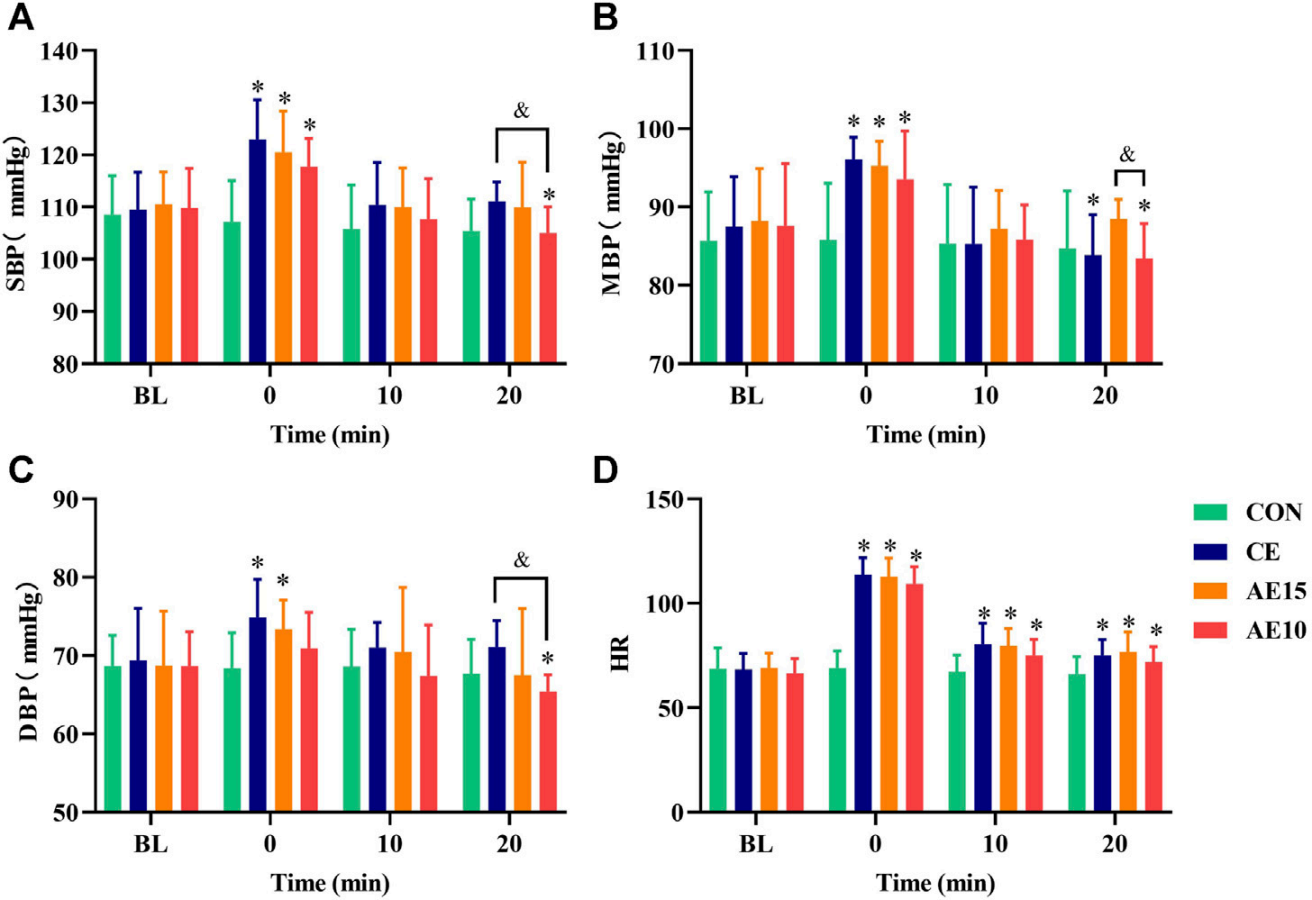
Effects of different exercise patterns on blood pressure and heart rate. **(A)** Systolic blood pressure (SBP). **(B)** Mean blood pressure (MBP). **(C)** Diastolic blood pressure (DBP). **(D)** Heart rate (HR). (*: *p* < 0.05 vs. BL. &: significant difference between trials. *p* < 0.05).

As demonstrated in Figures 5A–D, only diastolic pressure in AE10 did not significantly increase at 0 min. At 20 min, the systolic and diastolic pressures in AE10 were significantly lower than those at BL and in CE, and the mean blood pressure was significantly lower than that at BL and in AE15. At 0 min, the heart rate of all trials increased significantly and then decreased gradually.

**FIGURE 6:**
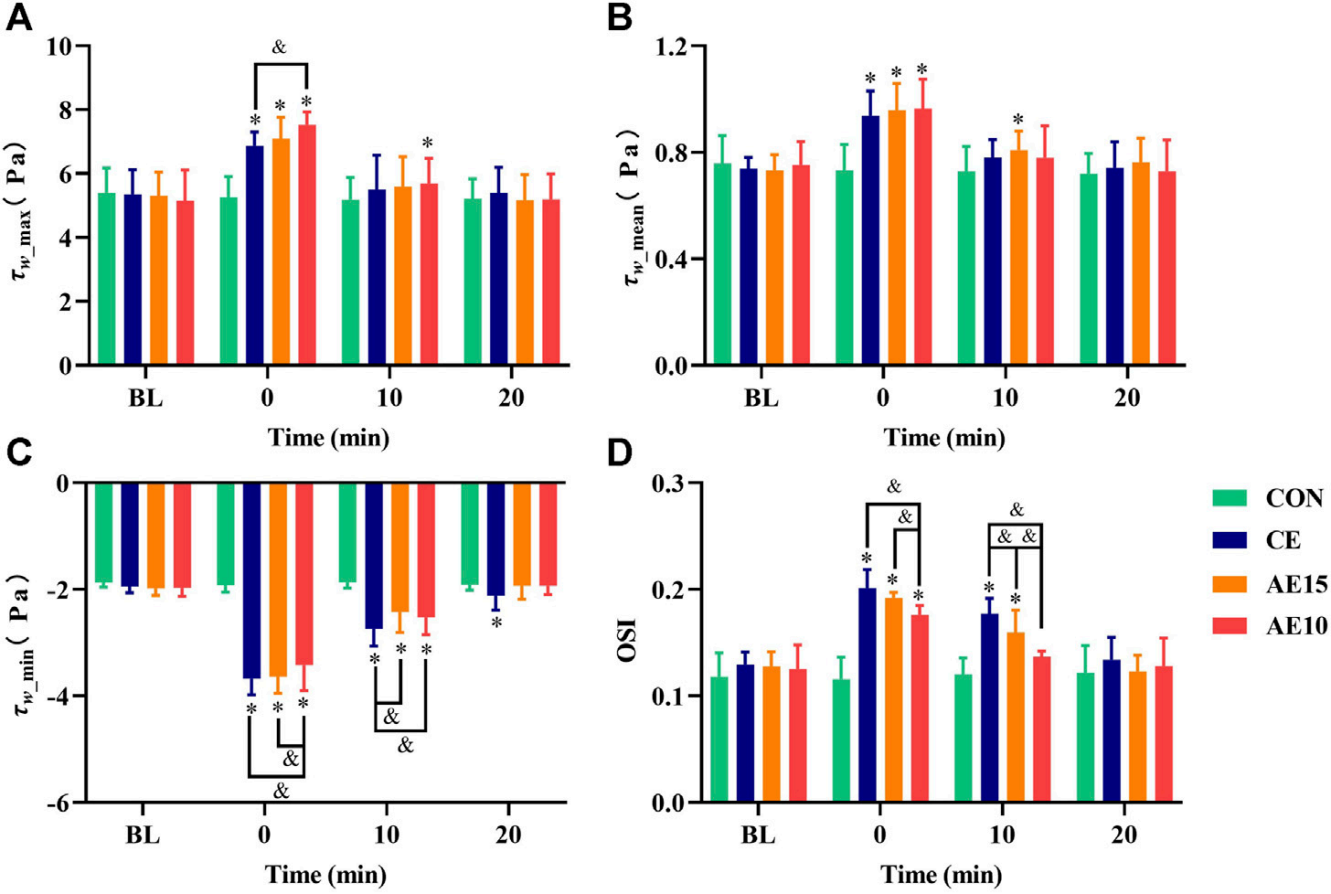
Effects of different exercise patterns on common carotid arterial wall shear stress and oscillatory shear indexes. **(A)** Maximum wall shear stress. **(B)** Mean wall shear stress. **(C)** Minimum wall shear stress. **(D)** Oscillatory shear index (OSI). (*: *p* < 0.05 vs. BL. &: significant difference between trials. *p* < 0.05).

As shown in Figures 6A–C, the maximal and mean wall shear stress ( $\tau_{w\_\max}$ and $\tau_{w\_\text{mean}}$ ) of all trials were significantly higher than that at the BL at 0 min, of which that in AE10 was the highest. In the meantime, the minimum wall shear stress ( $\tau_{w\_\min}$ ) of all trials had a negative value and significantly increased at 0 min, with the $\tau_{w\_\min}$ in AE10 being the lowest. The $\tau_{w\_\max}$ of AE10 was still significantly higher than that at the BL at 10 min. Figure 6D shows that the oscillatory shear index (OSI) of all trials was significantly increased; the OSI of AE10 is significantly lower than that of CE and AE15. At 10 min, the OSI of CE and AE15 was still significantly higher than that at BL and of AE10.

**FIGURE 7:**
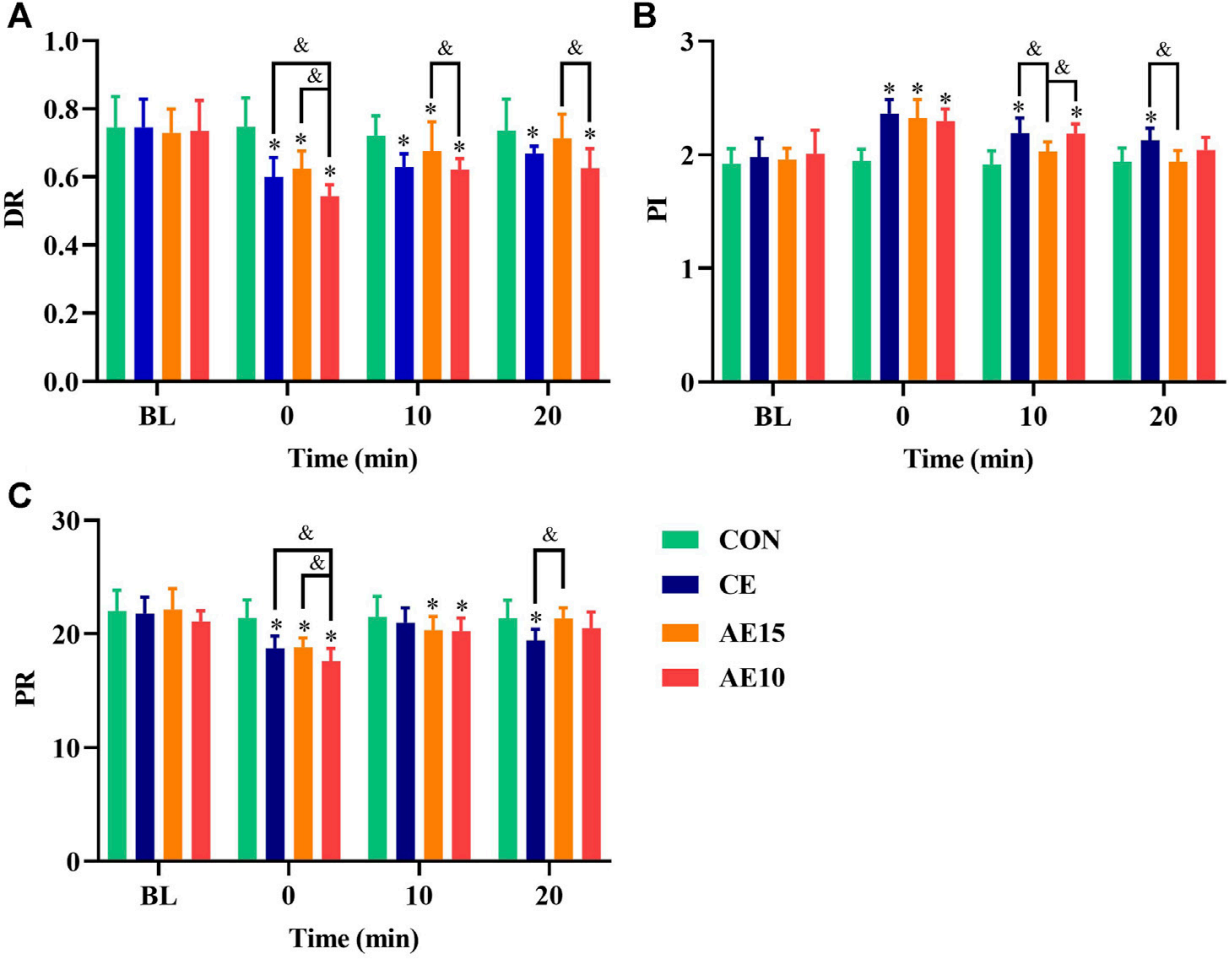
Acute effects of exercise on **(A)** carotid artery dynamic resistance (DR), **(B)** pulsatility index (PI), and **(C)** peripheral resistance (PR) at different intensities. (*: *p* < 0.05 vs. BL. &: significant difference between trials. *p* < 0.05).

Figure 7A shows that the dynamic resistance (DR) of all trials was significantly decreased at 0 min and that the DR of AE10 was the lowest. At 20 min, the DR of AE10 was still the lowest and significantly lower than that at BL. Figure 7B shows that the pulsatility index (PI) of all trials significantly increased at 0 min, and only the PI of CE did not completely recover at 20 min. Figure 7C indicates that the peripheral resistance (PR) of all trials was significantly decreased at 0 min, and the PR of AE10 was significantly lower than that of CE and AE15. However, only the PR of CE was still significantly lower than that at the BL at 20 min.

## 4 Discussion

Acute exercise exerts direct effects on the vasculature by the impact of exposure to hemodynamics stimuli, and prolonged regular exercise-induced hemodynamics changes induce the remodeling of vascular function and structure (Green et al., 2017). This study not only examines the effects of accumulated exercise and continuous exercise on arterial stiffness and hemodynamics but also compares the effects of accumulated exercise with different numbers of exercise bouts. The major findings are as follows: compared with continuous exercise and accumulated exercise with fewer bouts, accumulated exercise with more bouts has better acute effects on arterial stiffness and hemodynamics. This implies that an increased number of bouts can compensate for the shorter duration of each bout.

### 4.1 Response of arterial elastic functions to different exercise patterns

The buffering capacity of the artery is affected by its structural (wall thickness and composition), functional (stiffness), and geometric (diameter) characteristics (Farasat et al., 2008). Different types and intensities of exercise have different effects on arterial function. This study shows that the arterial stiffness (*β*) and elastic modulus (*E*_p_) of CE and AE15 were significantly increased at 0 min and recovered at 20 min. No significant changes were observed in AE10. Preceding studies have shown that the arterial stiffness immediately decreased after 30-min moderate-intensity accumulated exercise and did not recover for dozens of minutes (Zhou et al., 2015; Ren et al., 2022). This result is inconsistent with our result, which may be caused due to the different methods for evaluating arterial stiffness, such as brachial-ankle pulse wave velocity (baPWV) and cardiovascular-ankle vascular index (CAVI), and different body positions selected for detection.

To supply stable circulating blood to various organs of the human body, arterial elasticity is also regulated by the interaction between vasodilator and vasoconstrictor reflexes (controlled by sympathetic nerves) (Figueroa et al., 2019). Exercise leads to the increase of sympathetic excitation and then promotes the increase of arterial stiffness. An acute increase in inflammation and sympathetic nerve activation after high-intensity exercise may lead to a temporary increase in arterial stiffness. However, arterial stiffness may remain unchanged or decrease after low- to moderate-intensity exercise (Kresnajati et al., 2022). The increase in arterial stiffness in CE and AE15 at 0 min may be related to the increase in sympathetic excitation and hormone levels due to the intensity of exercise reaching a certain capacity. The arterial stiffness in AE10 has no obvious change that may be associated with the continuous self-adjustment of the body during repeated bouts.

At 0 min, the diameters of AE15 and AE10 were significantly lower than that of CE. A previous study has shown that the artery diameter did not obviously change immediately after moderate- to high-intensity continuous exercise (Naka et al., 2003), which is consistent with our study. The decrease in artery diameter may be related to vasoconstriction induced by the promotion release of endothelin-1, epinephrine (Greer et al., 1998), and catecholamine (Wray et al., 2007). Previous studies found that accumulated exercise results in lower postprandial glucose (PPG) (Zhang et al., 2022) and low-density lipoprotein cholesterol (Kelley et al., 2005) than continuous exercise. The decrease in arterial diameter in AE15 and AE10 was probably related to a similar mechanism.

### 4.2 Response of cerebral blood supply to different exercise patterns

The common carotid artery is the main channel for blood supply to the brain. Previous studies have found that aerobic exercise, resistance exercise, and integrated concurrent exercise could immediately increase blood supply to the brain (Wang et al., 2023), and high-intensity interval exercise might be beneficial for brain-related health as maintenance of cerebral perfusion in contrast to high-intensity continuous exercise (Tsukamoto et al., 2019). Our study shows that the *Q*_ma*x*_, *Q*_mean_, *V*_max_, and *V*_mean_ in all trials significantly increased at 0 min, in the following order AE10 > CE > AE15. At 10 min, only the *Q*_max_, *Q*_mean_, and *V*_max_ of AE10 were still significantly higher than those at the BL level. It is noteworthy that the *Q*_min_ values of all trials were negative and obviously higher than those at the BL at 0 min (CE > AE15 > AE10). The increase in the *Q*_min_ leads to the augmentation of blood flow fluctuation. The occurrence of the blood flow retrograde phenomenon may be related to the increase in heart rate and the significant shortening of the diastolic period after exercise, resulting in insufficient ventricular filling. It may also be due to the interaction of increased muscle sympathetic nerve activity (MSNA) (Padilla et al., 2010) and blood flow resistance of lower limbs (Casey et al., 2012).

To sum up, the AE10 trial has the most obvious effect on improving cerebral blood supply, and the blood flow fluctuation is smooth and lasts for a long time. Exercise can improve cerebrovascular function, cognition, and neuroplasticity through the increase in cerebral blood flow (Bliss et al., 2021). It can be inferred that the number of exercise bouts has greater effects on cerebral blood supply than the duration of a bout.

### 4.3 Response of hemodynamics to different exercise patterns

The SBP, MBP, and DBP of all exercise trials were immediately increased at 0 min, but only the increase of DBP in AE10 was not significant. Previous research has reported that diastolic blood pressure did not obviously change during or after exercise in normotensive subjects (Halliwill et al., 2013), and low-intensity exercise may inhibit sympathetic nerve excitation, reduce epinephrine secretion, and restrain vasoconstriction. On the contrary, high-intensity exercise may stimulate sympathetic nerve excitation, resulting in a muscle-derived response (Nobrega et al., 2014), which may lead to an increase in blood pressure immediately after exercise. At the same time, the blood pressure increased immediately after exercise, but the artery diameter decreased. This may be due to the activation of the vascular smooth muscle (VSM), which reduces the recruitment of collagen fibers and transfers the wall stress from collagen fibers to the VSM. This mechanism enables the vascular system to weaken the acute changes caused by the increase in blood pressure and maintain or even increase the buffering capacity of arteries (Santana et al., 2005). At 0 min, the increase in blood pressure in the CE group is more than that in AE15 and that in AE15 is more than that in AE10. This phenomenon may be related to the total exercise volume caused by the different intervals. However, following the exercise at 20 min, the SBP and DBP in AE10 were significantly lower than those at BL and in CE, and the MBP was significantly lower than that at BL and in AE15. A recent study summarized that a single bout of acute aerobic exercise reduces ambulatory BP over 24 h in adults (Saco-Ledo et al., 2021), which is consistent with our results. Our results show that accumulated exercise with more bouts has superior effects on decreasing blood pressure than the longer duration of a single bout.

Wall shear stress (WSS) is defined as the tangential friction force on the vascular wall during blood flow. WSS is important to vascular endothelial cell shape, size, function, and permeability (Chatzizisis et al., 2007). Lower WSS can induce endothelial dysfunction, lead to vascular wall thickening, enhance the migration of inflammatory cells to the arterial wall, and thus promote the process of atherosclerosis (Schlager et al., 2014). However, higher WSS in the normal range plays an important role in maintaining the normal function of the vascular endothelium (Gates et al., 2018). At 0 min, our results indicated that the wall shear stress ( $\tau_{w\_\max}$ and $\tau_{w\_\text{mean}}$ ) in all trials was significantly increased (AE10 > AE15 > CE), and the $\tau_{w\_\min}$ values in all trials were negative and significantly increased (absolute value: CE > AE15 > AE10). The oscillatory shear index (OSI) represents the proportion of reverse flow wall shear stress, and exacerbation of the oscillatory shear index may induce endothelial dysfunction (Storch et al., 2018). The OSI of all trials was significantly increased at 0 min, and a significant difference was observed in various trials (CE > AE15 > AE10). This difference lasted for at least 10 min. Considering WSS and OSI together, accumulated exercise with more bouts leads to higher WSS and lower OSI, which may have a more obvious effect on improving vascular endothelial function.

Dynamic resistance (DR) can reflect the arterial regulation ability, and a lower DR is beneficial to arterial regulatory capacity. Immediately after exercise, the DR of AE10 was significantly lower than that of BL, CE, and AE15, and at 20 min, the DR of AE10 was still the lowest and significantly lower than that at the BL. This means that accumulated exercise with more bouts has a more significant and lasting effect on reducing the DR. The pulsatility index (PI) indicates vascular compliance and blood flow elastic resistance of the arterial bed, and higher PI represents lower resistance. Peripheral resistance (PR) refers to the resistance received when blood flows to the periphery. In our results, the PI of all trials was significantly increased at 0 min, but only the PI of CE did not recover completely. Meanwhile, the PR of all trials decreased significantly at 0 min. Although the PR of AE10 was the lowest at 0 min, only the PR of CE was still significantly lower than that at the BL at 20 min. By and large, the effect of AE10 on blood flow resistance was more significant at 0 min, but the effect of CE was more lasting, and CE is more beneficial to improving the smoothness of blood flow in the common carotid artery.

Reducing fatigue is an important factor in urging people to persist in completing physical activities. Therefore, the results of this study support the use of accumulated exercise in more rounds to reduce the risk of cardiovascular diseases related to sedentary behavior. Moreover, the hemodynamics is not fully recovered during the interval. Compared with a single bout of continuous exercise, the total improvement time of hemodynamics is prolonged through the accumulation of multiple bouts, so the improvement of some indexes is more significant.

The superior mechanism of accumulated exercise on arterial stiffness and hemodynamics may be related to the bioavailability of nitric oxide (NO). NO is a gasotransmitter and regulator of myriad biochemical processes. The decrease in NO bioavailability with aging is especially apparent in sedentary individuals, whereas physically active individuals maintain higher levels of NO (Shannon et al., 2022). A previous study shows that in two continuous exercise tests with a 24-h interval, the plasma NO level increased slightly after the first exercise, while it increased greatly after the second exercise (Zdrenghea et al., 2003), which may be due to the accumulation of NO bioavailability in several bouts of exercise.

## 5 Conclusion

Generally speaking, our research results show that there is no significant difference between continuous exercise and accumulated exercise on the stiffness and diameter of the common carotid artery at moderate intensity. We also found that accumulated exercise plays a more significant role in improving cerebral blood supply, blood pressure, wall shear stress, and oscillatory shear index of the common carotid artery. The number of bouts has a more obvious impact than the duration of each bout. In improving blood flow resistance, both continuous exercise and accumulated exercise can produce favorable acute effects, but the former can produce more lasting improvement effects.

## 6 Limitations

This study also has limitations. First, we only recruited healthy young male participants; therefore, we cannot infer whether women and other populations have similar results. Second, we only analyzed the effects of different exercise patterns after the trial on arterial stiffness and hemodynamics, ignoring the monitoring of parameters during the trial. Third, we adopted the non-invasive method that used a formula with a form factor equal to 33% to calculate carotid pressure. Recent studies show that among the different non-invasive approaches used to calculate carotid pressure, the equation with the form factor equal to 33% showed the best association with the invasive measured (Bia et al., 2023b). However, when the blood pressure exceeds 130 mmHg, this calculation formula will increase the errors (Bia et al., 2023a). The blood pressure of all subjects was less than 130 mmHg during the course of our trial, so the results were reliable. Fourth, the subjects perform cycling while seated, but they have to take Doppler ultrasound measurements in a supine position, which may affect the results. Future research can better explore the mechanism of arterial stiffness and hemodynamics response after acute exercise intervention by controlling its limitations.

## Data Availability

The original contributions presented in the study are included in the article/Supplementary Material; further inquiries can be directed to the corresponding authors.
