# Supplementary material for: Effects of accumulated exercise on the stiffness and hemodynamics of the common carotid artery
Source: Front Physiol. 2024 Feb 26;15:1348811. doi: 10.3389/fphys.2024.1348811 (PMC10925650; doi:10.3389/fphys.2024.1348811)
Supplement: Supplementary file 1 [file DataSheet1.pdf]

Supplementary Material

Effects of Accumulated Exercise on the Stiffness and Hemodynamics of Common Carotid Artery

Haibin Liu[1,2*†], Bingyi Shen[3†], Zusheng Li[1], Chundong Xue[4], Hongling Zhao[2*], Xin Pan[2], Dong Xu[1]

*** Correspondence:** Corresponding Author: Haibin Liu, liuhaibin@dlut.edu.cn

Hongling Zhao, zhaohongling2000@126.com

1 Measurements and calculation of local hemodynamics

We measured the arterial inner diameter and center-line blood flow velocity waveforms of the right common carotid artery. In the meantime, heart rate, brachial systolic pressure (SBP) and diastolic pressure (DBP) were recorded on the left arm in triplicate by using a cuff-type electronic manometer. The same experimental method was described in detail in our previous research (Liu et al., 2018; Shen et al., 2020).

The detected arterial diameter waveform (Figure 2(A)) and center-line velocity (Figure 2(B)) were saved only as images. The self-compiled program in Matlab was used to extract blood vessel diameter (Figure 2(C)) and center-line velocity waveforms (Figure 2(D)). Heart rate signals were used to synchronize diameter and center-line velocity waveforms (beat-to-beat recording), then the carotid arterial blood pressure waveform (Figure 2 (E)) was calibrated using brachial pressure and arterial diameter waveforms.

The calibration formula is as follows:

1.1 Blood Pressure (*P*)

$$P_i = \frac{P_{\text{s\_mean}} - P_{\text{d\_mean}}}{D_{\max} - D_{\min}} \times (D_i - D_{\min}) + P_{\text{d\_mean}} \tag{1}$$

$P_{\text{s\_mean}}$ and $P_{\text{d\_mean}}$ are the systolic and diastolic pressures of the brachial artery, $D_{\max}$ and $D_{\min}$ are the max and min values of carotid artery diameter. D_i and P_i are the diameters and the calculated values of blood pressure at the same time. The maximum and minimum values of P_i are the calibrated SBP (P_{S}) and DBP (P_{d}), respectively.

We adopted the non-invasive method that used a formula with a form factor equal to 33% to calculate carotid pressure. Recent studies show that among the different approaches used to calculate carotid pressure, the equation that the form factor equal to 33% showed the best association with the invasive measured, when the SBP does not exceed 130mmHg (Bia et al., 2023b)(Bia et al., 2023a). The mean arterial pressure (P_{m}) was calculated by using the following equation:

$$p_{\mathrm{m}} = p_{\mathrm{d}} + \frac{1}{3}(p_{\mathrm{s}} - p_{\mathrm{d}}) \tag{2}$$

1.2 Flow Rate (*Q*)

The flow rate was calculated for

$$Q = 2\pi \bar{R_0}^2 \int_0^1 y \cdot u(y) dy \tag{3}$$

where R_0 is the average of the radius of the common carotid artery over time during a cardiac cycle.t is the period of one cardiac cycle. $y = r/R_0$, in which r is the radial coordinate. $u\,(y, t)$ satisfies:

$$u(y,t) = \sum_{n=-\infty}^{+\infty} \frac{J_0(\alpha_n j^{\frac{3}{2}}) - J_0(\alpha_n j^{\frac{3}{2}} y)}{J_0(\alpha_n j^{\frac{3}{2}}) - 1} u(0, \omega_n) e^{j\omega_n t} \tag{4}$$

where J_0 is the 0th-order Bessel function of the first kind and $j = \sqrt{-1}$. α_n is the Womersley number and n is the harmonic number.$\alpha_n = R_0 \sqrt{\rho \omega_n / n}$, in which ρ is the density of blood and η is blood viscosity. Due to the limited experimental conditions, n and ρ, in the present study, were taken as the same values for all subjects. n= 0.004 Pa·s and ρ = 1050 kg/m^3, respectively. $\omega_n = 2\pi n f$ is the angular frequency, and f is the base frequency. $u\ (0,\ \omega_n)$ is the n harmonic component of the measured center-line velocities. The maximal harmonic number n was computed as 20 and satisfies:

$$u(0,t) = \sum_{n=-\infty}^{+\infty} u(0, \omega_n) e^{j\omega_n t} \tag{5}$$

1.3 Apparent Elastic Modulus (*Ep*)

Arterial elastic function reflects the degree of change in arterial volume caused by changes in blood pressure per unit. The apparent elastic modulus was computed as:

$$E_p = \frac{p_s - p_d}{R_s - R_d} \cdot R_d \tag{6}$$

1.4 Apparent Stiffness Index (*β*)

β was calculated as the mean of adjusting arterial compliance for changes in normal stress as follows (Rossow et al., 2010):

$$\beta = \frac{ln\left(\frac{p_s}{p_d}\right)}{R_s - R_d} \cdot R_d \tag{7}$$

1.5 Wall Shear Stress (WSS)

The blood flowing along the vascular vessel creates a tangential frictional force, known as wall shear stress (τ_w), and was computed as:

$$\tau_w = \frac{\eta}{\bar{R}}\frac{\partial u}{\partial y}|y = 1 = \frac{\eta}{\bar{R}}\sum_{n=-\infty}^{+\infty}\frac{\alpha_n j^{\frac{3}{2}} J_1(\alpha_n j^{\frac{3}{2}})}{J_0(\alpha_n j^{\frac{3}{2}}) - 1} u(0, \omega_n) e^{j\omega_n t} \tag{8}$$

where J_1 is the first-order Bessel function of the first kind.

1.6 Oscillatory Shear Index (OSI)

The oscillatory shear index is an index that describes the ratio of the retrograde shear stress to the total shear stress and was defined as:

$$\text{OSI} = \frac{1}{2}\left(1 - \frac{\left|\int_0^T \tau_w dt\right|}{\int_0^T |\tau_w|\, dt}\right) \tag{9}$$

1.7 Pulsatility Index (PI)

The pulsatility index is used to express the relationship between blood flow pulsation and arterial pulsation and was calculated by the following equation:

$$\text{PI} = \frac{V_{\max} - V_{\min}}{V_{\text{mean}}} \tag{10}$$

where $V_{\max}$, $V_{\min}$, and V_{mean} are the maximum, minimum, and mean blood center-line velocities, respectively.

1.8 Dynamic Resistance (DR)

Dynamic resistance represents the ability of arterial regulation. The dynamic resistance was calculated as follows:

$$\text{DR} = \frac{p_{\text{s}} - p_{\text{d}}}{Q_{\max} - Q_{\min}} \tag{11}$$

where $Q_{\max}$ and $Q_{\min}$ are the maximum and minimum value of blood flow rate, respectively.

1.9 Peripheral Resistance (PR)

The peripheral resistance reflects the patency of capillaries in the brain and microcirculation in the intracranial vascular bed and was calculated by the following equation (Anderson and Phillips, 2015):

$$PR = \frac{p_{\mathrm{m}}}{Q_{\mathrm{mean}}} \tag{12}$$

2 Supplementary Figures

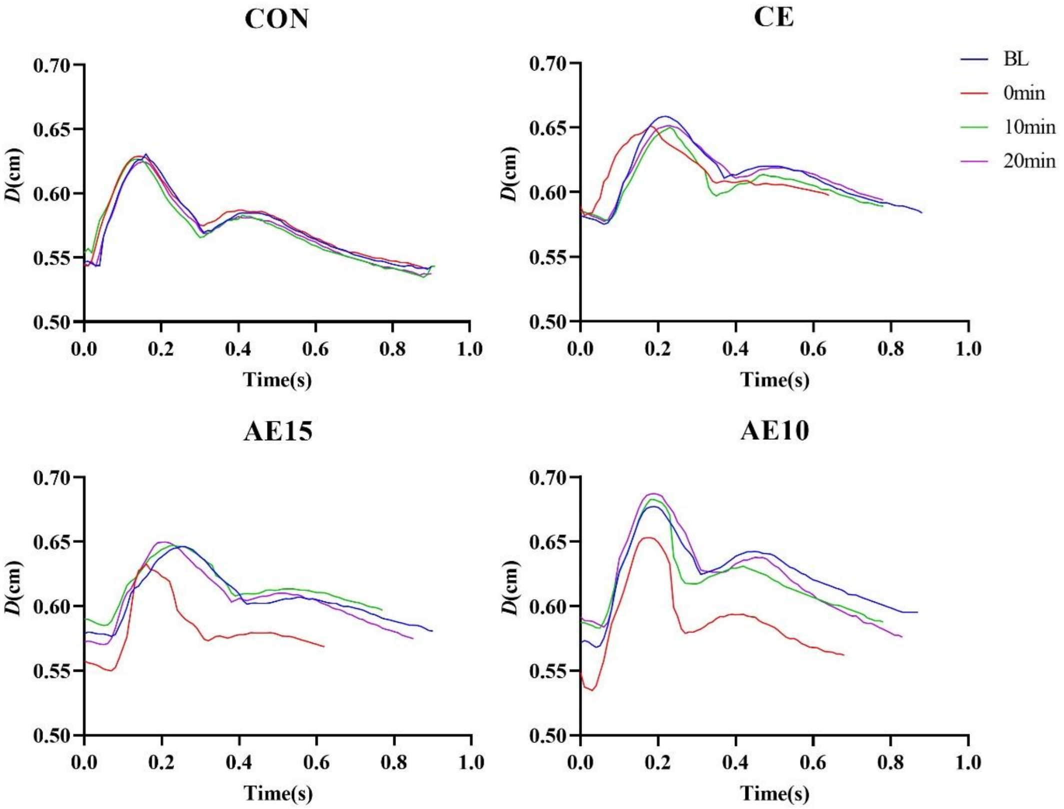

Supplementary Figure 1. The extracted waveforms of arterial inner diameter in different trials.

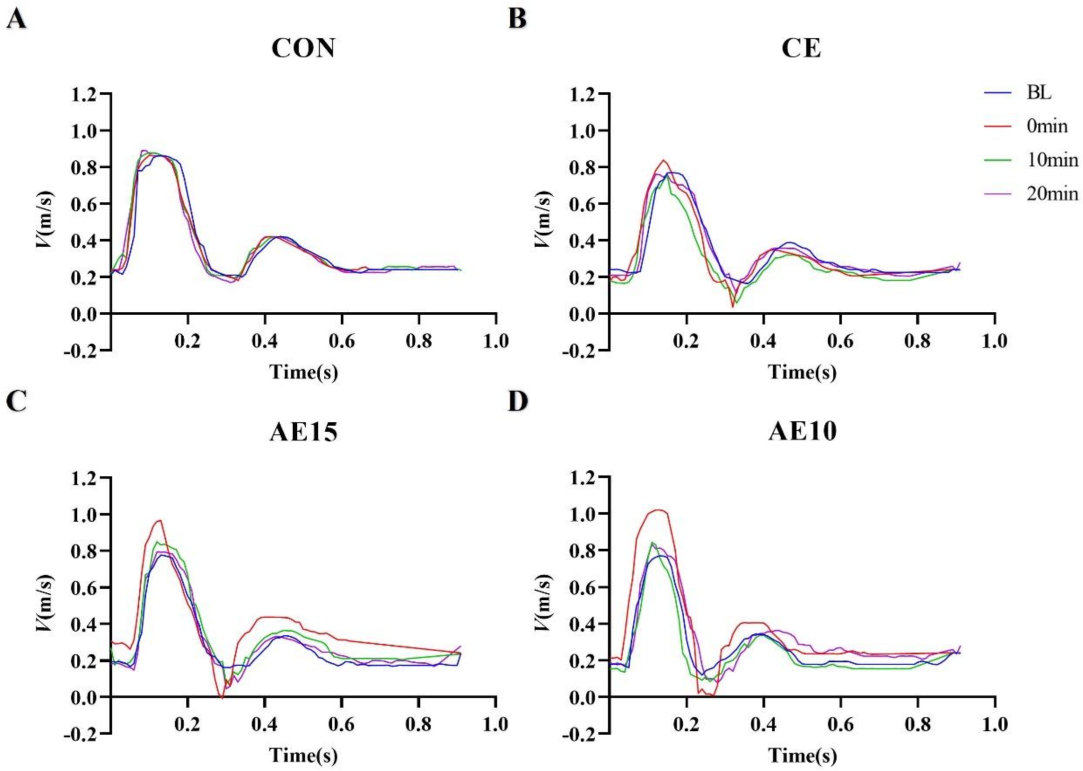

Supplementary Figure 2. The extracted waveforms of center-line blood flow velocity in different trials.

3 Reference

Anderson, T.J., and Phillips, S.A. (2015). Assessment and prognosis of peripheral artery measures of vascular function. *Prog Cardiovasc Dis* 57(5)**,** 497-509. doi: 10.1016/j.pcad.2014.11.005.

Bia, D., Zócalo, Y., Sánchez, R., Lev, G., Mendiz, O., Pessana, F., et al. (2023a). Aortic systolic and pulse pressure invasively and non-invasively obtained: Comparative analysis of recording techniques, arterial sites of measurement, waveform analysis algorithms and calibration methods. *Front Physiol* 14**,** 1113972. doi: 10.3389/fphys.2023.1113972.

Bia, D., Zócalo, Y., Sánchez, R., Torrado, J.F., Lev, G., Mendiz, O., et al. (2023b). Brachial Blood Pressure Invasively and Non-Invasively Obtained Using Oscillometry and Applanation Tonometry: Impact of Mean Blood Pressure Equations and Calibration Schemes on Agreement Levels. *J Cardiovasc Dev Dis* 10(2). doi: 10.3390/jcdd10020045.

Liu, H.B., Yuan, W.X., Wang, Q.Y., Wang, Y.X., Cao, H.W., Xu, J., et al. (2018). Carotid Arterial Stiffness and Hemodynamic Responses to Acute Cycling Intervention at Different Times during 12-Week Supervised Exercise Training Period. *Biomed Res Int* 2018**,** 2907548. doi: 10.1155/2018/2907548.

Rossow, L., Fahs, C.A., Guerra, M., Jae, S.Y., Heffernan, K.S., and Fernhall, B. (2010). Acute effects of supramaximal exercise on carotid artery compliance and pulse pressure in young men and women. *Eur J Appl Physiol* 110(4)**,** 729-737. doi: 10.1007/s00421-010-1552-1.

Shen, B.Y., Liu, H.B., Cao, L., and Qin, K.R. (2020). Acute Effects of Different Intensities of Cycling Acute Exercise on Carotid Arterial Apparent Elasticity and Hemodynamic Variables. *Biomed Res Int* 2020**,** 9027560. doi: 10.1155/2020/9027560.
